# Bone: Incretin Hormones Perceiver or Receiver?

**DOI:** 10.1155/2012/519784

**Published:** 2012-06-17

**Authors:** Ilaria Dicembrini, Edoardo Mannucci, Carlo Maria Rotella

**Affiliations:** ^1^Section of Endocrinology, Department of Clinical Pathophysiology, Careggi Teaching Hospital, University of Florence and Obesity Agency, 50127 Florence, Italy; ^2^Careggi Teaching Hospital, Diabetes Agency, 50127 Florence, Italy

## Abstract

Novel incretin-based drugs, such as glucagon-like peptide-1 receptor agonists (GLP-1 RA) and dipeptidyl peptidase-4 inhibitors (DPP-4i), have been last introduced in the pharmacological treatment of type 2 diabetes. In the last few years, the interest on the relationship of gut hormones with bone metabolism in diabetes has been increasing. The aim of present paper is to examine *in vitro* and *in vivo* evidence on the connections between incretin hormones and bone metabolism. We also discuss results of clinical trials and metaanalysis, explore the effects of incretin drugs *in vitro* on osteogenic cells and osteoclasts, and speculate on the possibility of different effects of GLP-1 RA and DPP-4i on the risk of bone fractures risk in humans. Although existing preliminary evidence suggests a protective effect on the bone, at least for DPP-4i, further controlled, long-term studies with measurement of bone markers, bone density, and clinical fractures rates are needed to substantiate and confirm those findings.

## 1. Introduction

Glucose, protein, and fat and mixed meal ingestion is associated with a significant reduction in markers of bone resorption, detectable by twenty minutes after feeding [1]. Bone formation is also influenced, but it seems to be less responsive to nutrients than resorption [2]. Biochemical assessment of bone turnover demonstrates that food intake is the major cause of the reduced bone turnover during daytime, which is followed by a nocturnal increase [3]. In addition, the observation that parenteral feeding is related to bone mass reduction [4] suggests a functional link between gut and bone metabolism through hormones responding to nutrients absorption, such as, incretins. The concept of incretins has been introduced to define gastrointestinal hormones released after meal ingestion, which modulate glucose homeostasis, mainly through both glucose-induced enhancement of insulin secretion and inhibition of glucagon release, such as glucagon-like peptide-1 (GLP-1). Beneficial extraglycemic actions on body weight, blood pressure, dyslipidemia, cardiac and endothelial function are further reported. Novel drugs based on the incretin system, such as, glucagon-like peptide-1 receptor agonists (GLP-1 RA) and dipeptidyl peptidase-4 inhibitors (DPP-4i), have been approved for the therapy of type 2 diabetes [5]. In the last few years, the interest on the relationship of gut hormones with bone formation and turnover in diabetes has been increasing, with preliminary data suggesting the possibility of positive effects of GLP-1 RA and DPP-4i on bone health. The aim of present paper is to examine in vitro and in vivo evidences on the connections between incretin hormones and bone metabolism. We also discuss results of clinical trials and meta-analysis, thus explore investigating the in vitro effects of incretin drugs in vitro on osteoblasts and osteoclasts, and speculate on the cells and presenting the possibility of different effects of GLP-1 RA and DPP-4i effects on the risk of bone fractures risk in humans clinical studies.

## 2. The Gut-Brain-Bone Axis and Diabetes

**Figure 1:**
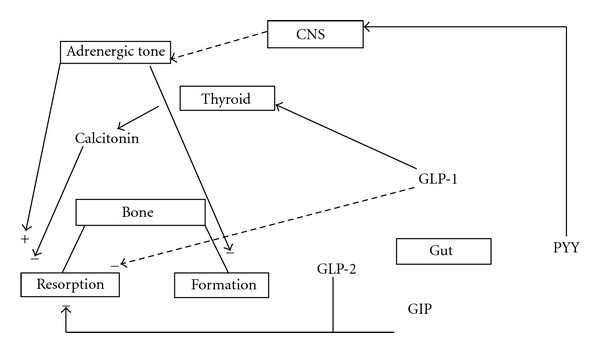
Gut mediators of the acute bone turnover in response to feeding. GLP-1: glucagon-like peptide-1; GLP-2: glucagon-like peptide-2; GIP: glucose-dependent insulinotropic peptide; PYY: peptide YY; CNS: central Nervous System. Broken lines represent putative pathways.

The regulation of bone turnover in response to feeding is complex with probable involvement of several mediators. The most important mediators identified are intestinal (GLP-1, GLP-2, Glucose-dependent Insulinotropic Peptide or GIP, and Peptide YY) and pancreatic beta cell (insulin, amylin, preptin, and pancreatic polypeptide) hormones [6]. Pancreatic peptides have direct actions on bone cells, while Peptide YY probably acts through arcuate nucleus in the central nervous system, thus regulating adrenergic tone and bone metabolism (Figure 1).

Diabetes is related to an increased risk of bone fractures [7]. A systematic review performed on 16 eligible studies indicates a significant increased risk of hip fracture both in type 2 diabetic women (overall relative risk (RR) 2.1; 95% confidence interval (CI): 1.3, 2.2) and men (overall RR 2.8; 95% CI: 2.6, 15.1) [8]. The observed increase in fracture risk is likely to be related to impaired bone quality rather than to bone mineral density. The related mechanisms, due at least in part to hyperglycemia, neuropathy, and higher incidence of hypovitaminosis D, are not yet fully understood [9]. However, disease progression is associated with low bone turnover, suggesting potential influences of antidiabetic agents on bone density and fracture rates. The increased incidence of bone fractures in patients with diabetes could also be due, at least part, to the effect of glucose- lowering therapies. It has been observed that long-term treatment with thiazolidinediones (TZDs) is associated with an increased risk of fracture in women with type 2 diabetes compared with other antidiabetic agents [10, 11]. The effect of TZD on bone fractures could be due to a specific inhibition of osteoblast differentiation and activity [12]. Furthermore, most available studies report a higher incidence of bone fractures in insulin-treated patients, in comparison with noninsulin-treated type 2 diabetic individuals [13], even after adjusting for concomitant antidiabetic medications [14]. The underlying mechanism is not completely understood, however, the contribution of an increased risk of falls induced by hypoglycemia cannot be excluded [15]. Moreover, a modifiable nutritional factor, such as, vitamin D deficiency, is also believed to play a role. Recent epidemiological evidence supports an increasing prevalence of hypovitaminosis D, inversely related to BMI, in all subset populations including children and adolescents [16]. Low 25-OH vitamin D levels are associated with higher probability of future diagnosis of type 2 diabetes, and in patients with established diabetes, with an increased incidence and progression of macro- and microvascular complications [17]. Cross-sectional studies confirmed an association between vitamin D status and risk of falls [18], but evidence from randomized clinical trials is required.

## 3. GLP-1 and Bone: Mechanisms of Action

GLP-1 is secreted by intestinal endocrine L cells, mainly after nutrient intake and rapidly inactivated by DPP-4 produced by endothelial cells. GLP-1 stimulates insulin secretion and inhibits glucagon secretion both in a glucose-dependent manner, thus ameliorating glucose homeostasis. A wide range of extrapancreatic actions on body weight, lipid profile and cardiovascular system has been recently described [5]. Mixed meal [19] and oral-glucose-load-induced [20] GLP-1 response have been reported to be reduced in Type 2 diabetes in comparison with healthy subjects; considering the possible involvement of GLP-1 in bone metabolism, the impairment of the GLP-1 axis could theoretically contribute to the increased risk of fractures in type 2 diabetes.

The actions of GLP-1 are predominantly mediated by a G protein-coupled receptor (GLP-1  R) expressed in the pancreas, stomach, intestine, kidney, lung, vascular system, heart, and brain. GLP-1  R activation stimulates adenylate cyclase, with formation of cyclic adenosine monophosphate (cAMP) and subsequent phosphorylation of protein kinase A [21]. In rodents, GLP-1  R has been detected on parafollicular thyroid C cells and GLP-1-mediated activation leads to C-cell proliferation and to calcitonin release, which could contribute to decrease bone resorption [22]. Moreover, genetic disruption of GLP-1 R in Glp-1 r^−/−^ knockout mice resulted in decreased cortical bone mass, and increased osteoclasts number. The bone resorption increase appeared to be sensitive to an acute calcitonin administration, thus promoting a calcitonin-dependent pathway in the GLP-1 mediated control of bone metabolism [23]. However, important differences on the expression levels of GLP-1 R between rodents and human have been described. In rodents (both mice and rats) C cells are relatively abundant, and calcitonin represents an important regulatory hormone in calcium homeostasis. In humans, conversely, C cells are significantly less represented and the physiological role of the hormone, except for some circumstances, such as, pregnancy and lactation, is uncertain [24]. Knudsen et al. showed a lack of functional response to GLP-1 in terms of cAMP production and calcitonin release in human TT thyroid C-cell line compared to rat C-cell lines MTC 6–23 and CA-77. The clinical relevance of these findings was confirmed by large clinical trials performed in type 2 diabetic patients treated with GLP-1 R agonists [22].

The possibility that GLP-1 might directly act on bone cells has also been investigated. The G protein-coupled GLP-1 R is expressed on human osteoblastic precursor cells [25] but not on mature osteoblasts [26]. The osteoblast activity modulation by GLP-1 seems to be related to different development stage. In human bone marrow stromal cells, GLP-1 promotes cellular proliferation and cytoprotection, preventing differentiation into adipocytes [27]. It has been recently demonstrated that GLP-1 can functionally interact with osteoblastic cells through a receptor, different from the GLP-1 R previously described. In liver and muscle [28], the effects of GLP-1 on glucose homeostasis are not related to a cAMP stimulation but to a rapid hydrolysis of glycosylphosphatidylinositoli (GIPs), generating inositolphosphoglycans (IPGs) and to a phosphatidylinositol-3 kinase (PI3K) and mitogen activated protein kinase (MAPK) activities. In a well-characterized later stage of osteoblastic cell line, such as MC3T3-EI, GLP-1 has shown to promote the immediate hydrolysis of GPIs, and this effect is consistent with the specific binding to a functional receptor independent of the cAMP-linked GLP-1 R. These data support the effect of IPGs as a second messenger and a GLP-1-induced stimulation upon PI3K and the existence of MAPK activities in osteoblastic cells [29] but required confirmation in vivo, particularly in humans.

In streptozotocin-induced diabetic and fructose-stimulated insulin-resistant rats, an insulin- and PTH-independent bone anabolic effect of GLP-1 has been recently shown, following 3-day continuous infusion on the trabecular bone structure [30]. In both these experimental models, GLP-1 and Exendin-4 (a natural GLP-1 RA) increased osteoprotegerin/receptor activated of NF-*κβ* ligand (OPG/RANKL) ratio, interacting with the Wnt pathway in osteoblasts to decrease bone remodeling. In particular, analysis of bone structure by microcomputer tomography supported a trend toward a small-size increase of BMD in the appendicular skeleton [31]. Similar results were reported in high-fat diet fed rats, following the same administration scheme [32]. These studies suggest a GLP-1-induced inhibition of bone resorption by osteoclasts, through direct effects on osteoblasts both in animal models of type 2 diabetes and metabolic syndrome, thus promoting a further careful evaluation of bone effects in ongoing Phase III clinical trials investigating the efficacy of a long-acting GLP-1 R analog, such as, liraglutide, in the treatment of obesity.

In response to feeding, as previously reported, different gut mediators are cosecreted. GIP, an incretin peptide, such as, GLP-1, is released from enteroendocrine K cells and functional GIP receptors are detected on osteoblasts-like cells, thus regulating their proliferation and activity. However, GIP receptors are in vitro downregulated by continuous exposure to GIP, thus requiring a pulsatile hormone release to stimulate osteoblasts [26]. Transgenic mice overexpressing GIP show increased bone mass and reduced bone loss with aging [33]. At the same time, GLP-2 and peptide YY are cosecreted with GLP-1 from L cells after feeding. GLP-2 receptors are expressed on osteoclasts, and a related decrease on bone resorption has been shown in vitro [26]. Peptide YY knock-out mice showed a significant decreased bone mass and a further increase of bone loss after ovariectomy [34].

## 4. Incretins and Bone:**** Clinical Evidence in Humans

Long-term exposure of type 2 diabetic patients to exenatide, an incretin mimetic agent, was not significantly associated to an increased bone fracture risk, despite the progressive weight loss: at 82 weeks an average weight reduction of 4.4 kg was reported, with a mean of 11.9 kg (−11.4% of baseline body weight) in highest weight loss quartile [35]. Several previously reported studies have shown that a 5–10% weight loss is associated to a significant decrease in bone mass and to an increase of bone resorption, especially in obese postmenopausal women [36]. Moreover, bone mineral density and markers of calcium homeostasis (serum alkaline phosphatase, calcium and phosphate) were not affected by 44 week treatment with exenatide in comparison to insulin glargine, a long-acting insulin, in type 2 diabetic subjects [37].

In a recent small double blind randomized clinical trial enrolling drug naïve type 2 diabetic patients, one-year treatment with DPP-4i (vildagliptin 100 mg daily) was not significantly related to significant change both in markers of bone resorption and calcium homeostasis in comparison to placebo [38].

A recent meta-analysis was performed including 28 clinical trials with a duration of at least 24 weeks, enrolling 11,880 and 9,175 patients for DPP-4i and comparators, respectively. Following a treatment of 35 weeks mean duration, 63 bone fractures were reported as serious adverse events. Despite short duration of trials, absence of discrimination between sex and pre-/postmenopausal state and evaluation of only severe bone fractures, DPP-4i, compared with placebo or other treatments, were associated with a reduced risk of fractures (Mantel-Haenszel odds ratio [MH-OR] 0.60, 95% CI 0.37–0.99, *P* = 0.045), even after the exclusion of comparisons with thiazolidinediones or sulfonylureas (MH-OR 0.56, 0.33–0.93, *P* = 0.026) [39].

On the other hand, GLP-2 injection in postmenopausal women resulted in a significant reduction of bone turnover in a dose-dependent manner [40]. The decrease of bone resorption by GLP-2 required an intact gastrointestinal tract, where GLP-2 receptors have been located in the myenteric plexus. The lack of GLP-2 response in jejunostomy patients [41] supported the afferent nerve fibres involvement in the regulation of bone metabolism by GLP-2.

## 5. Conclusions

The mechanisms through which feeding regulates bone turnover is complex and probably involved several mediators. Gastrointestinal peptides, such as, GLP-1, GIP, GLP-2 and peptide YY have been shown to favour bone formation over resorption. In the last few years, growing experimental evidences reported positive effects of novel incretin-based antidiabetic drugs on bone health. Clinical data on bone fractures risk profile during GLP-1 RA and DPP-4i therapies could vary with respect to their concomitant different (positive and neutral, resp.) effect on body weight. A positive action of GLP-1 RA on bone homeostasis could be overshadowed by weight loss-induced bone mass decrease, thus determining neutrality of GLP-1 RA treatment on bone fracture risk profile in human clinical trials. Moreover, despite stimulation of GLP-1 R through specific agonists, inhibition of incretin-hormone degrading enzyme DDP-4 enhances postprandial availability of different gut mediators of acute bone metabolism, such as, GLP-1, GIP, GLP-2, and peptide YY. Additional beneficial effects on bone health could be achieved by DPP-4i, in comparison to GLP-1 RA, through an overall involvement of the gut-brain-bone axis [6].

Taken together, this evidence could further explain potential different effects of GLP-1 RA and DPP-4i on bone fracture incidence and calcium homeostasis in human clinical studies. Further controlled, long-term studies with measurement of bone markers, bone density, and clinical fractures rates will be required to demonstrate conclusive efficacy along with underlying mechanisms responsible for incretin-related bone protection both in diabetic and not diabetic obese population. Pending further evidence, it is mandatory to promote the mainstay of osteoporosis prevention in type 2 diabetes: physically active, healthy lifestyle, and optimization of glucose control with low hypoglycemic risk, along with vitamin D repletion in deficient patients [42].
